# Natriuretic peptides appeared after their receptors in vertebrates

**DOI:** 10.1186/s12862-019-1517-x

**Published:** 2019-11-26

**Authors:** Anna Grandchamp, Shifa Tahir, Philippe Monget

**Affiliations:** 0000 0001 2182 6141 grid.12366.30 PRC, UMR85, INRA, CNRS, IFCE, Université de Tours, F-37380 Nouzilly, France

**Keywords:** Natriuretic peptide, Natriuretic peptide receptor, Phylogeny, Time of appearance

## Abstract

**Background:**

In mammals, the natriuretic system contains three natriuretic peptides, NPPA, NPPB and NPPC, that bind to three transmembrane receptors, NPR1, NPR2 and NPR3. The natriuretic peptides are known only in vertebrates. In contrast, the receptors have orthologs in all the animal taxa and in plants. However, in non-vertebrates, these receptors do not have natriuretic properties, and most of their ligands are unknown. How was the interaction of the NP receptors and the NP established in vertebrates? Do natriuretic peptides have orthologs in non-vertebrates? If so, what was the function of the interaction? How did that function change? If not, are the NP homologous to ancestral NPR ligands? Or did the receptor’s binding pocket completely change during evolution?

**Methods:**

In the present study, we tried to determine if the pairs of natriuretic receptors and their ligands come from an ancestral pair, or if the interaction only appeared in vertebrates. Alignments, modeling, docking, research of positive selection, and motif research were performed in order to answer this question.

**Results:**

We discovered that the binding pocket of the natriuretic peptide receptors was completely remodeled in mammals. We found several peptides in non vertebrates that could be related to human natriuretic peptides, but a set of clues, as well as modeling and docking analysis, suggest that the natriuretic peptides undoubtedly appeared later than their receptors during animal evolution. We suggest here that natriuretic peptide receptors in non vertebrates bind to other ligands.

**Conclusions:**

The present study further support that vertebrate natriuretic peptides appeared after their receptors in the tree of life. We suggest the existence of peptides that resemble natriuretic peptides in non-vertebrate species, that might be the result of convergent evolution.

## Background

**Fig. 1:**
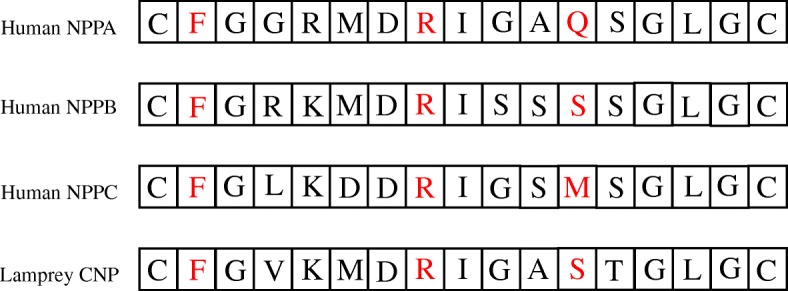
Amino acid sequences of the tree human natriuretic peptides and the lamprey natriuretic peptide. The amino acids involved in binding are shown in red

In mammals, the natriuretic system is composed of three peptides, the natriuretic peptide A named ANP, natriurtic peptide B (BNP) and natriuretic peptide C (CNP), also called NPPA, NPPB and NPPC, that bind to membrane receptors, NPR1, 2 and 3. ANP and BNP play a part in vasodilation. Natriuric peptide C induces the relaxation of smooth muscle cells on which it inhibits cell proliferation, and lacks natriuretic properties [1]. The natriuretic peptides were discovered thanks to their diuretic properties [2, 3]. They are also involved in other processes [4], such as cell proliferation, angiogenesis, apoptosis, inflammation [5], control of lipid metabolism [6], and meiotic arrest [7]. Each of the three peptides has cysteines separating 15 amino acids, and joined together to form a cysteine bridge, giving the peptide a loop structure (Fig. 1).

In other vertebrates, the natriuretic system mostly involves the same ligand and receptor families as in mammals. In fishes, all fish species do not have the same number of natriuretic peptides, and the system evolved during the teleost fish diversification [8]. Despite different evolutions, the natriuretic peptide NP remains predominant. In 2003, the hagfish’s CNP (ortholog of natriuretic peptides) was shown to be involved in natriuresis [9]. However, contrary to mammals, the natriuresis in fishes mainly gives rise to osmoregulation and the absorption of NaCL [10]. In amphibians, NPRC receptors were discovered in *Bufo marinus* [11] and several frog species [12], and were shown to interact with natriuretic peptide for the diuresis. In reptiles, the ANP peptides are missing. In birds, the BNP is involved in natriuresis [13], and the natriuretic peptide receptors are localised in the kidney in reptiles and birds [14]. Interestingly, other natriuretic peptides specifically appeared in some snake species [15]. They are called KNP, and are part of some snakes’ venom, as they still interact with the natriuretic peptide receptors of the preys.

The natriuretic peptides belong to a structurally related gene family [16]. They originated from a single gene, which in only known in vertebrates. A unique natriuretic peptide which seems to be phylogeneticaly related to CNP has been identified in hagfish [9]. In 2006, [17], identified a natriuretic peptide in several lamprey species, *Geotria australis*, *Lampetra japonica* and *Petromyzon marinus*. According to their work, these peptides would be more similar to CNP (CNP-4) than ANP and BNP, although the C-terminal tail of the sequence is missing. In Lamprey, the natriuretic peptide, is mostly involved in natriuresis, as in most of the other vertebrates [18] Apart from vertebrates, only one study attests to the possible existence of a natriuretic peptide in scorpion venom [19]. In the 1980s, the presence of natriuretic peptides was suggested in some mollusks and arthropods [20, 21]. However, these studies failed to sequence these potential peptides. Current literature agrees that the natriuretic peptides appeared in vertebrates [22].

The NPRA, NPRB and NPRC receptors (also called NPR1, NPR2 and NPR3) are transmembrane receptors with guanylyl cyclase (GC) activity. They are part of a family of 5 guanylyl cyclase receptors with Ret-GC-1 and Ret-GC-2 [2]. NPRC does not have intrinsic enzymatic activity. The NPRA, NPRB and NPRC receptors are encoded by paralog genes [23–26]. All are receptors for natriuretic peptides, with different binding affinities. The amino acids of the binding pockets of each of these receptors are specific. However, they share most of their binding amino acids [27].

Little is known about the activity of guanylyl cyclase receptors in non-vertebrate animals, and no substantial data indicate any part in sodium regulation or vasodilation. In echinoderms, the first Guanylyl cyclase receptors to have been isolated are involved in chemotaxis between egg and sperm [28]. In *C. elegans* which present a large expansion of the guanylyl cyclase receptor family, the majority of them are expressed in taste neurons [29]. No ligand is known in other protostomes.

There is a natriuretic system in insects, but it does not rely on the same ligands as the natriuretic system of vertebrates. The saline balance is managed by malpighian tubules and neurohormones [30, 31]. In the mosquito, the natriuretic peptide involved in sodium regulation is the Mosquito natriuretic peptide [32], which is an ortholog of vertebrate CRF (Corticotropin Releasing Hormone), [33], involved in stress response [34]. This peptide passes through cyclic AMP receptors (receptors for adenosine 5′-monophosphate) [33].

The phylogeny of natriuretic peptide receptors of vertebrate species is thus incongruent with that of their vertebrate ligands, which appeared later in the tree of life. This case is not isolated. [35] reported that more than 212 ligands appreared later than their recepor in the animal phylogeny. In 2012, [36] reworked the phylogeny of 15 peptide ligands with their receptors, to show that the phylogeny of ligands was more ancestral than expected, and that coevolution between ligands and their receptors was noted. A study of a large number of ligands and receptors conducted in 2013 by [37] led to the same observations.

Conversely, there are some cases of figures in which a ligand replacement has been made during evolution. For example, the FSHR receptor has an ortholog in protostomes [38], which binds to a FSH-like ligand, which is not an ortholog of FSH [39, 40].

In the case of the natriuretic peptide, one can hypothesize that an ancestral ligand (which may have disappeared) has given way to the natriuretic peptide of vertebrates, or that, on the contrary, an ortholog does indeed exist in the protostomes, too distant to be recognized by a simple Blast analysis. If such an ortholog exists, is it able to bind to the guanylyl cyclase receptors in these species, or has this interaction occurred only in vertebrates?

The natriuretic system is a system of choice to try to illuminate the evolutionary dynamics between a ligand-receptor couple. Here, we tried to answer the following questions:

- Would the receptors of protostomian species be able to bind to a vertebrate natriuretic peptide?
- Can any vertebrates natriuretic peptide be found in some non vertebrate species?
- According to the previous answers, what evolutionary dynamics have led to the establishment of the natriuretic system in vertebrates?

## Results

### Analysis of vertebrate pairs of ligand/receptor

**Fig. 2:**
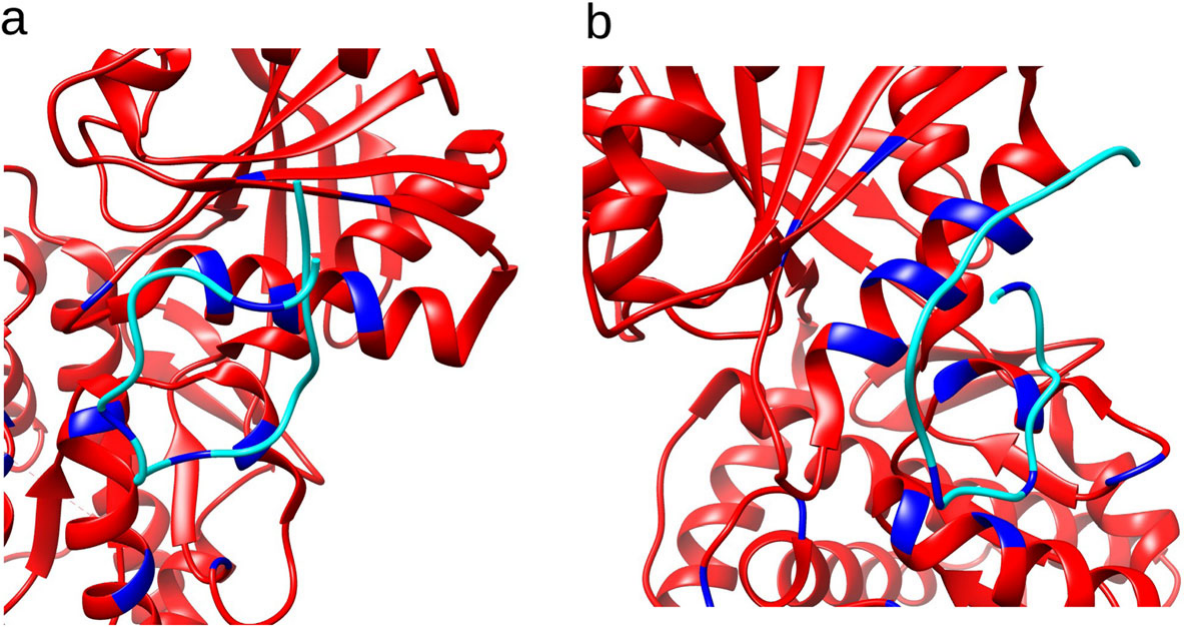
Binding of the natriuretic peptides with the **a** NPR1 and **b** NPR3 receptors retrieved from PDB structures 1yk0 and 1 t34. Receptors are in red, ligands in azul, and the amino acids involved in binding are in blue

The three natriuretic peptides bind to each of the 3 natriuretic peptide receptors with 3 highly specific residues (Fig. 1). Two of these three amino acids are present in all three peptides, namely the F phenylalanine (first position in the loop after the Cysteine) and the R Arginine (seventh position in the loop after the Cysteine). The third amino acid, in 11th position in the loop after the Cysteine, is different in the three peptides. It is a Glutamine for NPPA, a Serine for NPPB and a Methionine for NPPC. The binding of the peptides is made possible by the particular structure they share, namely two cysteines linked by a disulfide bridge, exposing a loop of 15 amino acids. The NPRC receptor binding pocket involves 12 different amino acids depending on the conformation of the ligand. The NPRA receptor binding pocket has 16 amino acids Fig. 2.

**Fig. 3:**
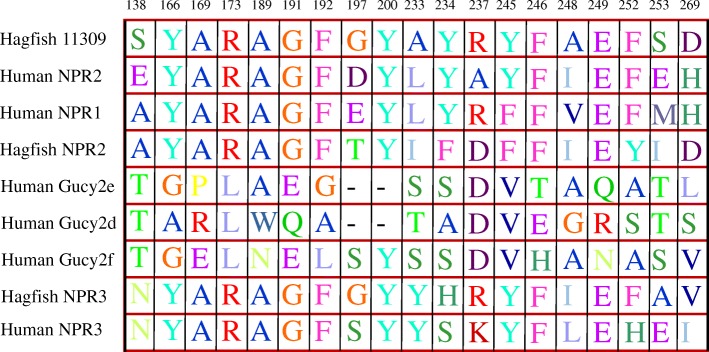
Alignment of the sequences of receptor binding pockets to human natriuretic peptide with that of hagfish. The numbers represent the position of the amino acids of the binding pocket in the human NPR1. The positions in the alignment are shown above each amino acid

The human natriuretic peptide receptors were aligned with the three receptors of sea lamprey and hagfish Fig. 3. The percentages of identity within the binding pocket vary between 50 and 75% (Table 1). This could indicate that perfect identity of the sequences of the binding pocket is not necessary to ensure binding to the natriuretic peptides. The same analysis was done with urochordates (Table 2) and protostomes (Table 3). In urochordates, *Apostichopus japonicus* and *Acantaster planci,* the binding pocket presents a hight percentage of amino acid similarity, with 6 to nine amino acids in common with human, despite it being less than hagfish. In protostomes, we found a maximum of 4 amino acids in common. Protostomes that are not present in the table have a percentage of identity of 0% at the place of the human binding pocket.

**Table 1:** Percentage of amino acids from the binding pocket of the natriuretic peptide receptors shared by Hagfish and human

Genes	Percentage
Hagfish unknown – human NPR1	9/15
Hagfish unknown – human NPR3	7/12
Hagfish NPR2 – human NPR1	9/15
Hagfish NPR2 – human NPR3	6/12
Hagfish NPR3 – human NPR1	7/15
Hagfish NPR3 – human NPR3	9/12

**Table 2:** Percentage of amino acids from the binding pocket of the natriuretic peptide receptors shared by chordate species and human

	Human NPR1	Human NPR3
*Ciona intestinalis*	5	5
*Hemichordata Saccoglosses*	1	2
*Apostichopus japonicus*	7	6
*Acantaster planci*	9	8
*Strongylocentrotus purpuratus*	0	0

**Table 3:** Number of amino acids from the binding pocket of the natriuretic peptide receptors shared by protostomian species and human

	Species
1 aa in common	*Zootermopsis nevadensis* *Nasonia vitripennis* *Trichinella spiralis* *Schistosoma mansoni* *Ixodes scapularis* *Daphnia pulex*
2 aa in common	*Strongylocentrotus purpuratus* *Caenorhabditis elegans* *Octopus bimaculoides*
4 aa in common	*Tetranychus urticae*

### Positive selection on guanylyl cyclase receptor binding pockets

According to our results, only one tree had indexes of positive selection: the phylogenetic tree of Protostomes, that included outgroup branches. In this unrooted tree, we observed that the protostomes species, as well as the outgroups, were mixed, and their evolution was not congruent with the tree of life. For example, chordates are grouped with protostomes. Similarly, the Paraneoptera (Protostoms) group has 3 species that cluster at opposite ends of our phylogeny. This fact demonstrates strong reaorganizations of the guanylyl cyclase receptors in non vertabrates species, and these changes seem to have occurred independently in each branch of the trees.

**Fig. 4:**
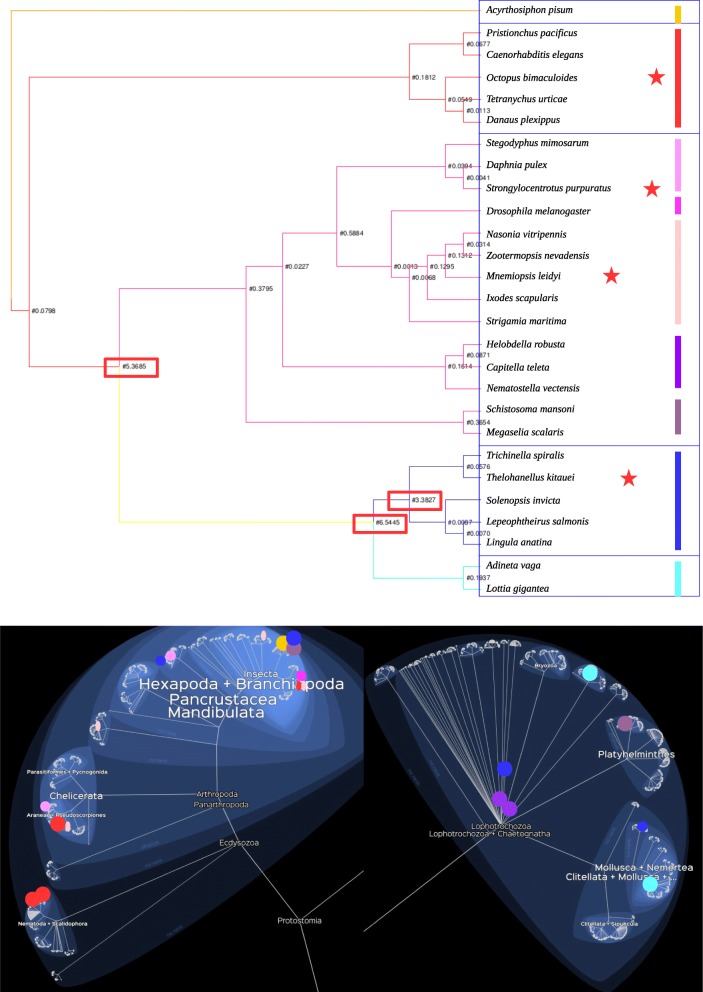
Phylogenetic tree of protostome (+ outgroups) guanylyl cyclase receptors. The red stars represent the outgroups. The groups generated by the tree are colored in the capture of lifemap

Three branches of this tree were found as being under positive selection (Fig. 4a and b). However, because the tree generated is not congruent with the tree of life, we suggest that the positive selection that we observe is biased, due to a resemblance in the peptides sequences between species that group together. We did not observe any positive selection in the other generated trees.

### Search of natriuretic peptides in non-vertebrates

**Fig. 5:**
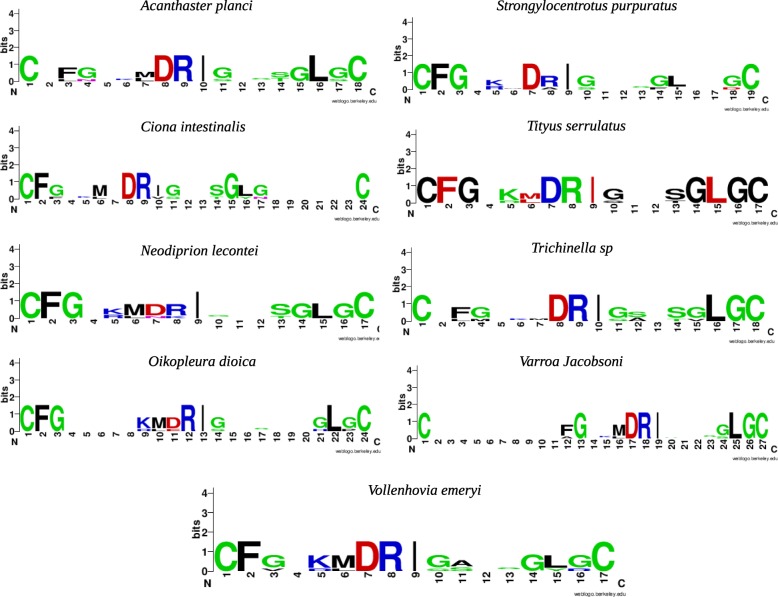
Logos of nine natriuretic peptides found in non-vertebrate animals

We searched for a potential natriuretic peptide in protostomes and in non-vertebrate chordates, using a regular expression search. We found 10 peptides in 9 organisms with a protein containing the considered motif (Fig. 5). Three of them are present in chordates (2 peptides were found in *A.planci*), and six in protostomes. We tried to determine if the potential 10 peptide sequences corresponded to one or several potential exons. The whole DNA sequences of each of the 10 proteins were tested with Genscan. All of these genes contained only one exon. These 10 proteins containing the peptides were aligned together. The alignments had very low identity scores (maximum of 10% ID between species), suggesting that these proteins don’t have a common origin.

A phylogenetic tree was built using RaxML, including the 10 peptides and NPPA, NPPB and NPPC coming from all the vertebrates branches. In the tree we got, all the 10 peptides were separated in different branches. Moreover, the bootstrap values of the tree were wery weak, indicating that the tree was not well supported (Additional file 1).

### Docking analysis

**Fig. 6:**
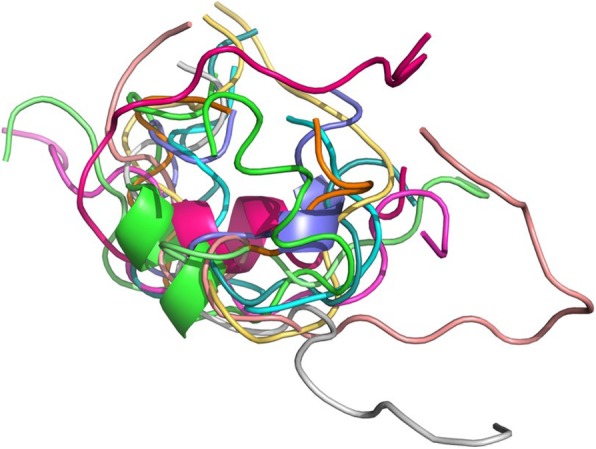
3D structure of the ten peptides found in non-vertebrate animals, whose protein sequence has similarities with that of human natriuretic peptides

**Fig. 7:**
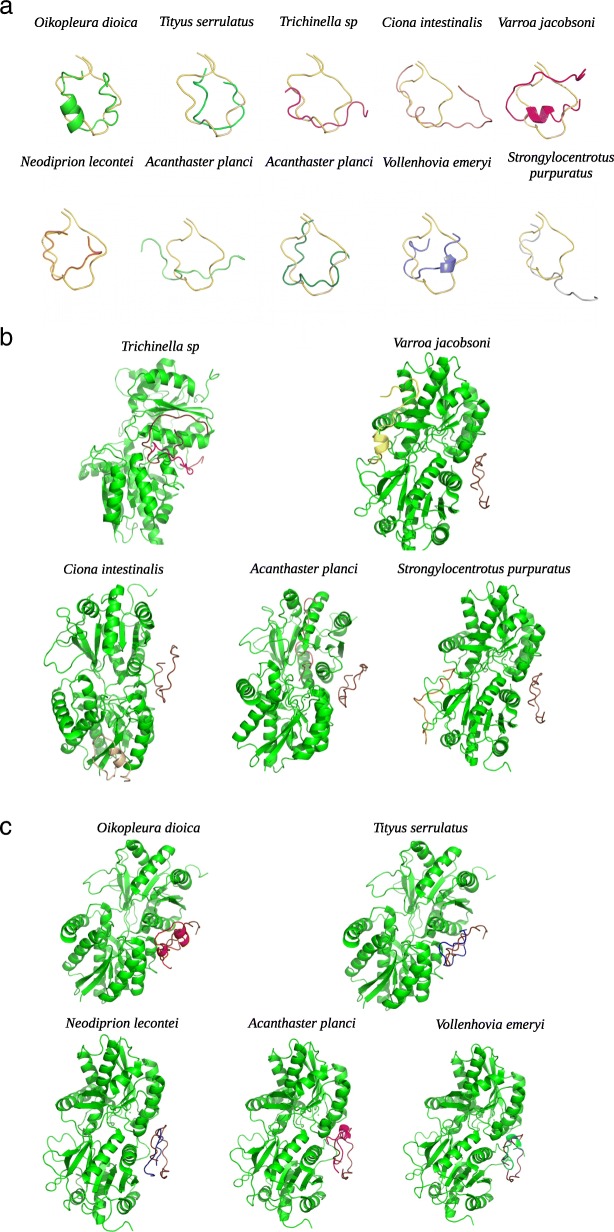
**a** Superposition of the three-dimensional structure of the human natriuretic peptide with the ten peptides found in non-vertebrate animals. The human natriuretic peptide is shown in yellow. **b** Model of binding of the five peptides from non-vertebrate animals in a binding pocket of the human NPR3 receptor different from that occupied by of human natriuretic peptides. The receptor is represented in green, the natriuretic peptide NPPA in brown. The five peptides from each of the five non vertebrate species are represented respectively in red, blue, violet, pink and azure. **c** Model of binding of the five peptides from non-vertebrate animals in the same binding pocket of the human NPR3 receptor as that occupied by the human natriuretic peptides. The receptor is represented in green, the natriuretic peptide NPPA in brown. The five peptides from each of the five non-vertebrate species are respectively represented in violet, yellow, beige, pink and orange

We retrieved potential natriuretic peptides from 10 non-vertebrate species. Then, the 3D structure of these 10 peptides was established as described in Mat & Meth section (Fig. 6). Interestingly, the 3D structure of these 10 peptides differs from that of the human natriuretic peptides (Fig. 7a). Despite this difference in sequence and 3D structure, these 10 peptides were docked with both human natriuretic peptide receptors. The results are the same for NPR1 and NPR3, so we only present the images of the NPR3 receptor. Of the 10 peptides found, five bind to the receptor in a binding pocket distinct from the binding pocket of human natriuretic peptides (Fig. 7b). Among them are three nonvertebrate chordates (*Ciona intestinalis, Acanthaster planci, Strongylocentrotus purpuratus*) and two protostomes (*Trichinella sp, Varroa jacobsoni*). The other five peptides bind to the natriuretic peptide receptor in the same binding pocket as the human natriuretic peptides (Fig. 7c). Of these five peptides, four belong to protostomes (*Oikopleura dioica, Tityus serrulatus, Neodiprion lecontei, Vollenhovia emeryi*) and one to a non-vertebrate chordate (*Acanthaster planci*). So, despite the absence of sequence similarity of 3D structure, these peptides from non vertebrate species seem to be able to bind to a binding pocket similar or not to that occupied by the human peptides. It bears noting the amino acids involved in the binding are not the same as the amino acids of the human natriuretic peptides, and are not located at the same positions.

### Vertebrate natriuretic system

We tried to determine how the vertebrate natriuretic system was built. The NPR3 receptor was modelled for several vertebrates, part of all the vertebrates tree, and docked with the 3 peptides. The main domain in all the selected vertebrate species have a 3D structure similar to the human’s one. The NPPA, NPPB and NPPC bound in the same binding pocket as the mammal one. In some species, the peptides bound with different conformations. However, this fact is also observed in human [41]. Our results confirmed that all of the vertebrates have the same natriuretic peptide binding. We also modelled the NPR3 receptor ancestral to the vertebrates. By docking, we also observed that the 3 peptides bound in the same binding pocket as mammals but with different conformations (Additional file 1).

The different NPR3 and NPR1 binding pockets were also analysed in a set of fishes and coelacanthes. For NPR3, the percentages of similarity of the binding pockets were very high in all of the vertebrates. Even in fishes that diverged 420 ma from mammals, the percentage ID of the binding pocket was 83% (Additional file 1), while the percentage ID of the all sequence was 55% on average. In contrast, the NPR1 binding site is not as well conserved in mammals, with only 50% of ID in some fishes.

## Discussion

In this study, we tried to understand if the pairs of natriuretic peptide receptors and their ligands come from an ancestral pair, or if the interaction only appeared in vertebrates. We realized that the natriuretic peptide receptor binding pocket had been extensively remodeled during evolution. In addition, the phylogeny of protostomes has allowed us to see that important sequence rearrangements have occurred in receptors of natriuretic peptides, specifically within each species. In fact, the phylogenies are not congruent with the animal tree of life, suggesting that the receptors diverged markedly and specialised in each species. Some of these rearrangements are likely to be under positive selection. However, it should be noted that applying positive selection tests to such phylogenetically distant groups remains questionable.

Secondly, we looked for potential natriuretic peptides in the protostomes. We found 10 potential candidates. Among them, we found the known scorpion natriuretic peptide. However, a set of tests and arguments lead us to conclude that these sequences do not function as natriuretic peptide, and that their resemblance is incidental.

### Modification of the binding pocket

Alignment of human natriuretic peptide receptor with hagfish receptor demonstrated that the sequence similarities of the binding pocket vary from 50 to 75%. It seems that not all amino acids of the binding pocket are necessary for the binding. In fact, lampreys, hagfish and humans receptors all bind to natriuretic peptides, whereas we have shown that they do not share all of their amino acids. The human natriuretic peptide receptors were aligned both with chordates, and with several protostomians species. The alignments of human natriuretic peptide receptors with chordate receptors revealed partial amino acid identity of the binding pocket. For example, *Acanthaster planci* shares nine amino acids with the binding pocket of human NPR1 and eight with NPR3. *Apostichopus japonicus* shares seven amino acids with the human NPR1 binding pocket, and 6 with the human NPR3 binding pocket. These similarities may indicate that these two species could potentially bind with a natriuretic peptide, that is not identified in chordates. The binding of a receptor to its ligand may be dependent on the entire binding pocket, or may be limited to a restricted number of essential amino acids. For example, NMDA receptors possess three amino acids essential for binding, while three other amino acids in the binding pocket affect affinity, but their mutation may not invalidate the interaction [42]. A study conducted on class B GPCRs [43] shows that among these receptors, some amino acids are common to all receptors, while others are variable, and may be divergent while being involved in the binding to the ligand. Regarding the shared receptors, [44] demonstrate that several ligands can bind with the same affinity on the N-terminal domain of the GLP-1 receptor, suggesting different molecular interactions. Also, the changes of some amino acids in the binding pockets have a greater effect on the affinity constant than on the ability to bind. Moreover, mutagenesis experiments made it possible to observe several ranges of receptor binding affinity, for example in the case of fibronectin binding domains [45]. Nevertheless, there are also case studies for which the change of a single amino acid can invalidate or systematically be causative of the binding. For example, the Influenza virus has been shown to bind to two different receptors when only one of its amino acids is changed [46].

On the contrary, the other chordates as well as the protostomians species do not share strong sequence identities with the human receptors binding pockets. This suggests that the binding pocket is not highly conserved. The protostome and other chordates receptors probably do not possess the human ligand binding pocket. However, in our study, we only investigated the conservation of the binding pocket in an alignment. Yet, the conservation of the amino acids identity is not the only factor in composing a binding site. The surface, depth, and form of the binding site play an important role [47]. Moreover, different amino acids with similar physico-chemical properties can conserve a binding site [48]. Unfortunately, 3D structures of the guanylyl cyclase of these species do not exist, and their sequence identity with vertebrates receptors was too weak to allow the construction of a 3D structure. Our results cannot attest with certainty whether these receptors can bind to natriuretic peptides.

However, a second argument suggests that the binding pocket was remodelled during evolution. The phylogenetic trees of protostomians and chordate appeared to be unreliable. Species that are phylogenetically distant were grouped together and closely related species were separated. Moreover, we found that some branches were under strong positive selection. This suggests that the sequence of the non-vertebrate animals changed quickly in the close taxa. These elements suggest that the receptor could have different ligands in each branch of the tree of life.

We tried to determine whether the genes coding for a natriuretic peptide were present in protostomes and chordates. Ten candidates were identified. Nevertheless, several indications tend to suggest that they are not orthologous to the vertebrate natriuretic peptides. First of all, the alignments of the 10 peptides show very low scores, indicating that the coding genes seem not to be homologs with one another. Moreover, the sequences corresponding to the potential natriuretic peptides were part of larger proteins. But no potential splicing was detected between an exon and an intron, strongly suggesting that the natriuretic peptide included in a protein is never isolated. It is of course possible that the peptides are cleaved by an enzyme in vivo. However, if such is not the case, this would suggests that a binding between the natriuretic peptide-like part of the protein and the receptor requires, in their 3D structure, the exposition at the surface of this peptide part. If this is the way the system works, its functioning is not the same as that of the human natriuretic peptides, that are cleaved and work as peptides.

The peptides were found by motif search. None of the sequences corresponding to the peptides were 100% identical to the human peptide sequences. In order to find out whether these potential peptides would have the same three dimensional structure as the human ones, we generated models of them and analysed the 3D geometry. Then, a docking test was performed with these 10 peptides and the human natriuretic peptide receptor C. The structure modeling results revealed that the 3D structures of these peptides were different from one another, and from the human peptides. Our docking results indicate that these peptides do not bind to human receptors in the same way and place as human natriuretic peptides.

These clues have lead us to suggest that the 10 peptides found in non-vertebrate animals cannot be called natriuretic peptides, and do not perfom the same binding with the natriuretic receptors. It may be that their slight closeness in sequence to the human natriuretic peptides is fortuitous, rather than an ancestral relationship. If the 10 peptides are not spliced (indicating that they are only part of biggest proteins, and never produced as peptides) and are not homologues to natriuretic peptides, what are their functions? To get an idea, each peptide was submitted to a BLAST analysis in GenBank, to search for potential orthologs with a known function. Among the 10 peptides, two were part of proteins that already had a new function. In *Brazilian Scorpion*, the peptide is liberated with toxins, and acts as a vasodilator. In fact, the protein doesn’t act as an endogenous peptide, and does not possess associated receptors in this species. *Ciona intestinalis* protein also had a known function. The entire protein is an enzyme, involved in cell survival and groth. The protein is likely not clived. Concerning the eight remaining proteins: the BLAST for *Strongylocentrotus purpuratus* and *Oikopleura dioica* didn’t give significant result, as no one homolog was identified. In contrast, the other BLAST identified possible homologs. Most of them were enzymes, supporting the fact that the peptide we found are not clived, and are part of proteins being enzymes (Additional file 1).

In other gene families, there are several cases where ancestral ligands have been found in organisms where their receptors were present. For example, GNRH is a neuropeptide found only in vertebrates [49]. Nevertheless, a set of studies on its phylogeny have made it possible to detect GNRH orthologs in molluscs and other protostomes able to bind to their receptors, already known in these species. Although the function has diverged, the gene encoding the ligand was as ancestral as its receptor.

Many studies describe cases of incidental similitudes between peptides. Notably, venom peptides often show such similitudes [50]. This is the case, for example, with the venom present in platypus, whose peptide composition is similar to the venom of certain snakes, like-genes encode defensin-like peptides [51]. This is also the case with anurans. An antimicrobial peptide (AMP) is similar in 3 lineages, Neobatrachia, Bombinatoridae and Pipidae. Nevertheless, one study showed that this similarity resulted from an evolutionary convergence between the genes encoding these peptides [52]. In 2000, [53], reported a set of studies attesting random similitudes between genes coding for peptides.

In the present study, it is likely that there has been a random emergence of proteins whose part of the sequences is seemingly similar to that of the natriuretic peptides in protostomes. Interestingly, this is not the first such case of the appearance of proteins similar to natriuretic peptides. Some plants seem to possess a system that can be related to the natriuretic system of vertebrates. The natriuretic peptide of plants, PNP, also bind to guanylyl cyclase type receptors. In *Arabidopsis thaliana*, the natriuretic peptide AtPNP-A causes a modification of the water content of the cells, causing a regulation of cell volume [54]. Nevertheless, the resemblance between the plant natriuretic peptide and that of vertebrates might be the result of chance, rather than of any orthology relationship. In fact, the *Arabidopsis* gene coding for the natriuretic peptide is a very close homolog to another plant gene coding for expansin [55]. Also, some of these genes will have undergone an evolutionary convergence in *A.thaliana* to produce a natriuric peptide [56].

### Natriuretic system in vertebrates

How did the natriuretic system appeare in vertebrates? No NP homolog gene was found in the current nonvertebrate species, so the potential existence of a natriuretic peptide coding gene is unlikely in these species. The information that we obtained tend to indicate that the binding to NPR3 with the natriuretic peptide appeared in the ancestor of the vertebrates. Indeed, it seems that the GC receptors developed their vertebrate binding pocket independently from the existence of the peptide.

In vertebrates, NP have several functions, as described in the introduction. Particularly, it has been described as playing a part in the acrosome reaction during gamete fecundation [57]. Yet, this function in reproduction was also described in Echinodermata, for chemotaxis between egg and sperm [28]. Moreover, we demonstrated that the rGC (receptor guanylyl cyclase) of Echinoderms have a binding pocket quite similar to the vertebrates’ one. Therefore, we can suggest that the first function of the natriuretic peptide of vertebrates was involved in reproduction. The appearance of new functions and remodelling of the binding pocket, moreover happened several times in rGC evolution,. In *Caenorhabditis elegans*, GCY-9 was suggested to be involved in the detection of environmental CO2 [58], but other rGC are involved in taste neurons [29]. In the fruit fly *Bactrocera dorsalis*, the rGC BdmGC-1B was described to be involved in the reception of the EH (eclosion hormone), as also suggested in *Manduca sexta, Bombyx mori* and *Drosophila melanogaster* [59]. In arthropods, some rGC were described as being involved in the reception of chemicals and gustation [60]. Finally, in the Crustacean *Callinectes sapidus*, some have hypothesised that a rGC was the receptor for the molt-inhibiting hormone (MIH) [61].

The NPR1 binding pocket, however, is seemingly different in vertebrates that diverged first from the mammalian branch. Our data can suggest that the NPR1 binding with natriuretic peptides took place in a second time.

## Conclusions

In essence of the present research we can firmly say that the natriuretic peptides found in protostomes are not orthologs to the vertebrate ones, and that natriuretic peptide receptors originated earlier in evolution than their vertebrates ligands.

## Methods

### Analysis of vertebrate pairs of ligand/receptor

The available PDB structures of the human natriuretic peptide receptors, NPR1 and NPR3, were retrieved from PDB (with PDB ids: 1yk0 and 1 t34 respectively). The amino acids of the NPR1 and NPR3 receptors involved in binding the NPPA, NPPB and NPPC ligands were identified through literature search [62].

We investigated if the receptor’s binding pockets of the lamprey and hagfish, that are the vertebrates most distant to mammals, possessed homologous amino acid sequences to human receptors. The protein sequences of the three human natriuretic peptides and the three receptors were retrieved from Ensembl [63]. Using ensembl phylogenetic trees, the orthologs of the human natriuretic peptide receptor were identified and retrieved in the lamprey and hagfish.

Human receptors were aligned with lamprey and hagfish otholog receptors with MUSCLE software [64]. The human amino acids involved in the binding of ligands were compared with those of these two agnates, and the percentage of identity between amino acids involved in the binding pocket was calculated Fig. 2.

### Analysis of the binding pockets of chordates and protostomes receptors

We then investigated if, in non-vertebrate species, the GC receptors (that are orthologs of natriuretic peptide receptors) had the same binding pocket as vertebrates, or if the binding site was not present in these species.

The GC receptors of 23 protostomes species were obtained from Ensembl metazoa. These species were selected to sample the widest possible phylogenetic range of protostomes (Additional file 1).

To obtain the urochordate guanylyl cyclase receptors sequences, we searched in GenBank [65] by using tBLASTn and BLASTp. Five receptor sequences were retrieved in the following taxonomic groups representing non-vertebrate chordates: Enterogona, Hemichordata, Echinodermata, Aterozoa and Echinoidea.

We did two alignments using Muscle; one using Human, Lamprey, hagfish, and urochordate receptors, the other with Human, Lamprey, hagfish and protostome receptors. The percentage identity of the amino acids involved in binding between human and non vertebrates receptor was calculated.

### Positive selection on guanylyl cyclase receptor binding pockets

We investigated if the natriuretic receptors were under the influence of positive selection during animal evolution. Because the current protostome and deuterostomes diverged more than 600 ma ago, we suspected that the selection indexes could be biaised. Indeed, we built several trees in order to group together taxonomic groups. One tree was built with protostomes only, another with protostomes + urochordates, 1 with *C. elegans*, one with urochordates + vertebrates, and one with vertebrates alone. To test the congruence of the resulting protostome tree, we tried a RaxML unrooted tree with added two ourgroup species of first metazoans and two of chrodates species.

To accomplish the aforementioned tasks, both amino acid and nucleotide sequences of the receptors were considered. Amino acid sequences were aligned taking into account nucleotide alignment with Pal2Nal software [66]. Phylogenetic trees were generated with RAxML software [67]. Positive selection tests were conducted with the CodeML software from PAML [68]. The selection by branch was determined with the model 2 with no clock. A site-based selection was also conducted. To perform the codon based analysis, we used unrooted trees and selected the option: no clock in the tree. The F3X4 codon matrix was used, and we performed the one ratio-model (model = 0), and NS sites fixed at: one w, nearlyNeutral, and positive selection.

### Search of natriuretic peptides in non-vertebrates

We investigated whether the natriuretic peptide could be found in non-vertebrate animals. As described in the introduction, the human natriuretic peptides have a common motif. We performed psi_BLAST on GenBank to find peptides corresponding to the same motif as human natriuretic peptides in non-vertebrates species. We used the following regular expression. C - (12–18) X -C, by which a motif of 12 to 18 amino acids surrounded by 2 cysteines were searched.

### Docking

We checked whether the peptides found in non-vertebrate species corresponded to the same motif as human natriuretic peptides, and were also able to bind to guanylyl cyclase receptors. To answer this question, we performed a docking of potential peptides (ranging 17 to 25 amino acids) with vertebrate receptor, using CABS-dock [69]. CABS-dock works in 4 steps:

1: Docking simulation of a fully flexible peptide and a flexible protein receptor using the CABS model: docking simulation starts from random conformation of a peptide placed in a random position around the protein receptor structure. 2: Filtering of the models based on CABS protein peptide interaction energy values 3: Clustering and scoring of the final models. 4: Reconstruction of the final models to all-atom representation.

### Appearance of the binding system in vertebrates

We tried to understand which of the receptor or the ligand of the natriuretic system first acquired the ability to bind its partners. First, we modelled the 3D structure of the partners in all vertebrates to see if the binding was the same in all the branches. Homology modelling was performed to model the 3D structure of NPR3 receptor. All the templates selected for modelling came from human receptors, because only they came as putative hits. The % identity between the query sequences and templates ranges between 56 to 77%. Dto and absenceof templ, was not model, to avoid any bias.

Secondly, we generated the vertebrate ancestral sequence of NPR1 and NPR3. The sequences were generated using a representative set of all the vertebrates sequences, and the FASTML software [70] using JTT model and gamma distribution. The NPR1 ancestral sequence was modelled and docked with NPPA, NPPB and NPPC.

**Fig. 8:**
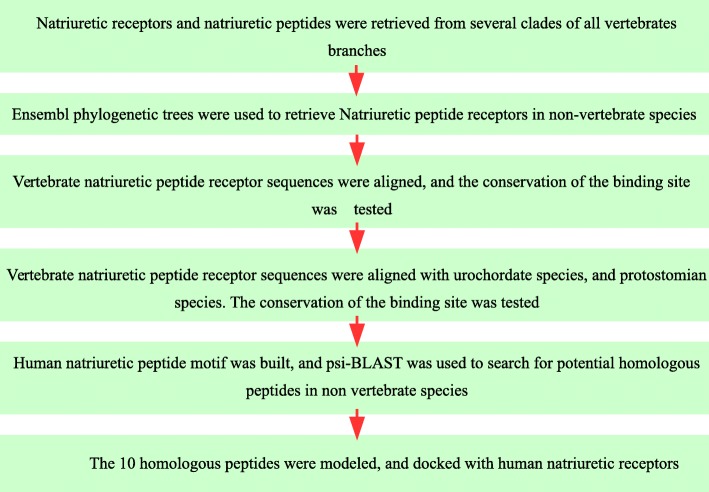
The flowchart summarizes the principal steps followed in the methodology of the article. The order by which the works were conducted is indicated by the arrows

Thirdly, the sequences of the mammal NPR3 and NPR1 receptor and teleost that earliest diverged (fishes and coelacanthes) were aligned, and the binding sites were analysed. A summary draw of the main steps of the method is shown in Fig. 8

.

## Supplementary information

**Additional file 1.** Supplemental figures and datas of the tests mentionned in the article.

## Data Availability

The data are available in the article, and in the supplemental datas.

## Abbreviations

NPPA: Natriuretic peptide A
NPPB: Natriuretic peptide B
NPPC: Natriuretic peptide C
NPR1: Natriuretic peptide receptor 1
NPR2: Natriuretic peptide receptor 2
NPR3: Natriuretic peptide receptor 3

## References

[1] Mercadier J-J (2005). Peptides Natriurétiques : Aspects Physiopathologiques. MT Cardio.

[2] Kisch B (1956). Electron microscopy of the atrium of the heart. I. Guinea Pig. Exp Med Surg.

[3] Henry JP, Pearce JW (1956). The Possible Role of Cardiac Atrial Stretch Receptors in the Induction of Changes in Urine Flow. The Journal of Physiology.

[4] Volpe M, Rubattu S, Burnett J (2014). Natriuretic Peptides in Cardiovascular Diseases: Current Use and Perspectives. European Heart Journal.

[5] Rubattu S, Sciarretta S, Valenti V, Stanzione R, Volpe M (2008). Natriuretic Peptides: An Update on Bioactivity, Potential Therapeutic Use, and Implication in Cardiovascular Diseases. American Journal of Hypertension.

[6] Bordicchia, Marica, Dianxin Liu, Ez-Zoubir Amri, Gerard Ailhaud, Paolo Dessì-Fulgheri, Chaoying Zhang, Nobuyuki Takahashi, Riccardo Sarzani, Sheila Collins. Cardiac Natriuretic Peptides Act via P38 MAPK to Induce the Brown Fat Thermogenic Program in Mouse and Human Adipocytes. The Journal of Clinical Investigation 122, no 3 (mars 2012): 1022‑1036. 10.1172/JCI59701 PMC3287224 22307324

[7] Zhang M, You-Qiang S, Sugiura K, Xia G, Eppig JJ (2010). Granulosa Cell Ligand NPPC and Its Receptor NPR2 Maintain Meiotic Arrest in Mouse Oocytes. Science (New York, N.Y.).

[8] Inoue JG, Miya M, Tsukamoto K, Nishida M (2003). Basal Actinopterygian Relationships: A Mitogenomic Perspective on the Phylogeny of the ‘Ancient Fish. Molecular Phylogenetics and Evolution.

[9] Kawakoshi A, Hyodo S, Yasuda A, Takei Y (2003). A single and novel natriuretic peptide is expressed in the heart and brain of the most primitive vertebrate, the Hagfish (*Eptatretus burgeri*). J Mol Endocrinol.

[10] McCormick SD (2001). Endocrine Control of Osmoregulation in Teleost Fish. Integrative and Comparative Biology.

[11] Meier SK, Toop T, Donald JA (1999). Distribution and characterization of natriuretic peptide receptors in the kidney of the toad, Bufo Marinus. Gen Comp Endocrinol.

[12] Kloas W, Hanke W (1992). Atrial natriuretic factor (ANF) binding sites in frog kidney and adrenal. Peptides.

[13] Takei Y (2000). Structural and Functional Evolution of the Natriuretic Peptide System in Vertebrates. International Review of Cytology.

[14] Brenner D, Gerstberger R (1999). Functional Receptors in the Avian Kidney for C-Type Natriuretic Peptide. Endocrinology.

[15] Sridharan, Sindhuja, and R Manjunatha Kini. “Snake Venom Natriuretic Peptides: Potential Molecular Probes.” BMC Pharmacology & Toxicology 16, no. Suppl 1 (2015): A87

[16] Potter LR, Abbey-Hosch S, Dickey DM (2006). Natriuretic peptides, their receptors, and cyclic guanosine monophosphate-dependent signaling functions. Endocr Rev.

[17] Kawakoshi A, Hyodo S, Nozaki M, Takei Y (2006). Identification of a Natriuretic Peptide (NP) in Cyclostomes (Lamprey and Hagfish): CNP-4 is the ancestral gene of the NP family. Gen Comp Endocrinol.

[18] Toop T., Grozdanovski D., Potter I. C. (2008). Natriuretic peptide binding sites in the gills of the pouched lamprey Geotria australis. Journal of Experimental Biology.

[19] Alves RS, Ximenes RM, Jorge ARC, Nascimento NRF, Martins RD, Rabello MM, Hernandes MZ (2013). Isolation, homology modeling and renal effects of a C-type natriuretic peptide from the venom of the Brazilian Yellow Scorpion (Tityus Serrulatus). Toxicon.

[20] Vesely David L (2013). Cardiac hormones for the treatment of cancer. Endocrine-Related Cancer.

[21] Chen AC (1989). Demonstration of a Human Atrial Natriuretic Peptide-Like Material in the Stable Fly, Stomoxys Calcitrans. Insect Neurochemistry and Neurophysiology.

[22] Takei Y (2000). Structural and functional evolution of the natriuretic peptide system in vertebrates. Int Rev Cytol.

[23] Drewett JG, Garbers DL (1994). The Family of Guanylyl Cyclase Receptors and Their Ligands. Endocrine Reviews.

[24] Koller KJ, Goeddel DV (1992). Molecular Biology of the Natriuretic Peptides and Their Receptors. Circulation.

[25] Potter LR, Hunter T (2001). Guanylyl Cyclase-Linked Natriuretic Peptide Receptors: Structure and Regulation. The Journal of Biological Chemistry.

[26] Silberbach, M., et C. T. Roberts. Natriuretic Peptide Signalling: Molecular and Cellular Pathways to Growth Regulation. Cellular Signalling 13, no 4 (april 2001): 221‑231. 10.1016/s0898-6568(01)00139-5 11306239

[27] He X-l, Dukkipati A, Garcia KC (2006). Structural Determinants of Natriuretic Peptide Receptor Specificity and Degeneracy. Journal of Molecular Biology.

[28] Singh S, Lowe DG, Thorpe DS, Rodriguez H, Kuang WJ, Dangott LJ, Chinkers M, Goeddel DV, Garbers DL (1988). Membrane Guanylate Cyclase Is a Cell-Surface Receptor with Homology to Protein Kinases. Nature.

[29] Ortiz CO, Etchberger JF, Posy SL, Frøkjaer-Jensen C, Lockery S, Honig B, Hobert O (2006). Searching for Neuronal Left/Right Asymmetry: Genomewide Analysis of Nematode Receptor-Type Guanylyl Cyclases. Genetics.

[30] Schooley DA, Horodyski FM, Coast GM (2012). 9 - Hormones Controlling Homeostasis in Insects. In Insect Endocrinology, 366‑429.

[31] Coast G (2007). The Endocrine Control of Salt Balance in Insects. General and Comparative Endocrinology.

[32] Coast GM, Garside CS, Webster SG, Schegg KM, Schooley DA (2005). Mosquito Natriuretic Peptide Identified as a Calcitonin-like Diuretic Hormone in Anopheles Gambiae (Giles). The Journal of Experimental Biology.

[33] Beyenbach KW (2003). Transport Mechanisms of Diuresis in Malpighian Tubules of Insects. The Journal of Experimental Biology.

[34] Timpl P, Spanagel R, Sillaber I, Kresse A, Reul JM, Stalla GK, Blanquet V, Steckler T, Holsboer F, Wurst W (1998). Impaired Stress Response and Reduced Anxiety in Mice Lacking a Functional Corticotropin-Releasing Hormone Receptor 1. Nature Genetics.

[35] Grandchamp, Anna, and Philippe Monget. “Synchronous Birth Is a Dominant Pattern in Receptor-Ligand Evolution.” BMC Genomics 19, no. 1 (2018): 611. 10.1186/s12864-018-4977-2 PMC6092800 30107779

[36] Mirabeau O, Joly J-S (2013). Molecular Evolution of Peptidergic Signaling Systems in Bilaterians. Proceedings of the National Academy of Sciences.

[37] Jékely G (2013). Global view of the evolution and diversity of metazoan neuropeptide signaling. Proc Natl Acad Sci U S A.

[38] Tatar M, Bartke A, Antebi A (2003). The Endocrine Regulation of Aging by Insulin-like Signals. Science.

[39] Simonet G, Poels J, Claeys I, Van Loy T, Franssens V, De Loof A, Vanden Broeck J (2004). Neuroendocrinological and Molecular Aspects of Insect Reproduction. Journal of Neuroendocrinology.

[40] De Loof A, Baggerman G, Breuer M, Claeys I, Cerstiaens A, Clynen E, Janssen T, Schoofs L, Vanden Broeck J (2001). Gonadotropins in Insects: An Overview. Archives of Insect Biochemistry and Physiology.

[41] Potter LR, Hunter T (2001). Guanylyl Cyclase-Linked Natriuretic Peptide Receptors: Structure and Regulation. The Journal of Biological Chemistry.

[42] Kuryatov A, Laube B, Betz H, Kuhse J (1994). Mutational analysis of the glycine-binding site of the NMDA receptor: Structural similarity with bacterial amino acid-binding proteins. Neuron.

[43] Hoare SRJ (2005). Mechanisms of peptide and nonpeptide ligand binding to Class B G-protein-coupled receptors. Drug Discovery Today.

[44] de Maturana L, Rakel AW, Kuntzsch A, Rudolph R, Donnelly D (2003). The Isolated N-Terminal Domain of the Glucagon-like Peptide-1 (GLP-1) Receptor Binds Exendin Peptides with Much Higher Affinity than GLP-1. The Journal of Biological Chemistry.

[45] Hackel BJ, Kapila A, Wittrup KD (2008). Picomolar Affinity Fibronectin Domains Engineered Utilizing Loop Length Diversity, Recursive Mutagenesis, and Loop Shuffling. Journal of Molecular Biology.

[46] Glaser L, Stevens J, Zamarin D, Wilson IA, García-Sastre A, Tumpey TM, Basler CF, Taubenberger JK, Palese P (2005). A Single Amino Acid Substitution in 1918 Influenza Virus Hemagglutinin Changes Receptor Binding Specificity. Journal of Virology.

[47] Nayal M, Honig B (2006). On the Nature of Cavities on Protein Surfaces: Application to the Identification of Drug-Binding Sites. Proteins: Structure, Function, and Bioinformatics.

[48] Valdar WSJ, Thornton JM (2001). Protein–Protein Interfaces: Analysis of Amino Acid Conservation in Homodimers. Proteins: Structure, Function, and Bioinformatics.

[49] Tsai P-S, Zhang L (2008). The Emergence and Loss of Gonadotropin-Releasing Hormone in Protostomes: Orthology, Phylogeny, Structure, and Function. Biology of Reproduction.

[50] Casewell NR, Wüster W, Vonk FJ, Harrison RA, Fry BG (2013). Complex cocktails: the evolutionary novelty of venoms. Trends Ecol Evol.

[51] Whittington CM, Papenfuss AT, Bansal P, Torres AM, Wong ESW, Deakin JE, Graves T (2008). Defensins and the Convergent Evolution of Platypus and Reptile Venom Genes. Genome Research.

[52] König E, Olaf RP (2011). Bininda-Emonds. Evidence for Convergent Evolution in the Antimicrobial Peptide System in Anuran Amphibians. Peptides.

[53] Kay BK, Kasanov J, Knight S, Kurakin A (2000). Convergent evolution with combinatorial peptides. FEBS Lett.

[54] Turek I, Gehring C (2016). The Plant Natriuretic Peptide Receptor Is a Guanylyl Cyclase and Enables CGMP-Dependent Signaling. Plant Molecular Biology.

[55] Kende H, Bradford K, Brummell D, Cho H-T, Cosgrove D, Fleming A, Gehring C (2004). Nomenclature for Members of the Expansin Superfamily of Genes and Proteins. Plant Mol Biol.

[56] Wang YH, Donaldson L, Gehring C, Irving HR (2011). Plant natriuretic peptides. Plant Signaling & Behavior.

[57] Revelli A, Ghigo D, Moffa F, Massobrio M, Tur-Kaspa I (2002). Guanylate cyclase activity and sperm function. Endocr Rev.

[58] Carrillo MA, Guillermin ML, Rengarajan S, Okubo RP, Hallem EA (2013). O2-Sensing Neurons Control CO2 Response in C. Elegans. Journal of Neuroscience.

[59] Chang J-C, Yang R-B, Adams ME, Kuang-Hui L (2009). Receptor guanylyl cyclases in inka cells targeted by eclosion hormone. Proc Natl Acad Sci.

[60] Byrne JH. The oxford handbook of invertebrate neurobiology: Oxford University Press; 2019.

[61] Zheng J, Lee C-Y, Watson RD (2006). Molecular Cloning of a Putative Receptor Guanylyl Cyclase from Y-Organs of the Blue Crab, Callinectes Sapidus. General and Comparative Endocrinology.

[62] Bartels CF, Bükülmez H, Padayatti P, Rhee DK, van Ravenswaaij-Arts C, Pauli RM, Mundlos S (2004). Mutations in the Transmembrane Natriuretic Peptide Receptor NPR-B Impair Skeletal Growth and Cause Acromesomelic Dysplasia, Type Maroteaux. The American Journal of Human Genetics.

[63] Aken BL, Achuthan P, Akanni W, Amode MR, Bernsdorff F, Bhai J, Billis K (2017). Ensembl 2017. Nucleic Acids Res.

[64] Edgar RC (2004). MUSCLE: Multiple Sequence Alignment with High Accuracy and High Throughput. Nucleic Acids Research.

[65] Benson DA, Cavanaugh M, Clark K, Karsch-Mizrachi I, Lipman DJ, Ostell J, Sayers EW (2013). GenBank. Nucleic Acids Research.

[66] Suyama M, Torrents D, Bork P (2006). PAL2NAL: robust conversion of protein sequence alignments into the corresponding codon alignments. Nucleic Acids Res.

[67] Stamatakis A (2014). RAxML version 8: a tool for phylogenetic analysis and post-analysis of large phylogenies. Bioinformatics.

[68] Yang Z (2007). PAML 4: Phylogenetic Analysis by Maximum Likelihood. Molecular Biology and Evolution.

[69] Kurcinski M, Jamroz M, Blaszczyk M, Kolinski A, Kmiecik S (2015). CABS-Dock Web Server for the Flexible Docking of Peptides to Proteins without Prior Knowledge of the Binding Site. Nucleic Acids Research.

[70] Ashkenazy H, Penn O, Doron-Faigenboim A, Cohen O, Cannarozzi G, Zomer O, Pupko T (2012). FastML: a web server for probabilistic reconstruction of ancestral sequences. Nucleic Acids Res.

