# Supplementary material for: Natriuretic peptides appeared after their receptors in vertebrates
Source: BMC Evol Biol. 2019 Nov 26;19:215. doi: 10.1186/s12862-019-1517-x (PMC6878697; doi:10.1186/s12862-019-1517-x)
Supplement: Supplementary file 1 [file 12862_2019_1517_MOESM1_ESM.odt]

Part 1. Protostomians species

Acyrthosiphon pisum

Pristionchus pacificus

Caenorhabditis elegans

Tetranychus urticae

Danaus plexippus

Stegodyphus mimosarum

Daphnia pulex

Drosophila melanogaster

Nasonia vitripennis

Zootermopsis nevadensis

Ixodes scapularis

Strigamia maritima

Helobdella robusta

Capitella teleta

Nematostella vectensis

Schistosoma mansoni

Megaselia scalaris

Trichinella spiralis

Solenopsis invicta

Lepeophtheirus salmonis

Lingula anatina

Adineta vaga

Lottia gigantea

Part 2 Phylogenetic tree 

(((((((20chinese_turtle_NPPB:0.64242115,21xenopus_nppb:0.78105222)0.5789:0.10551734,19platypus_NPPB:1.06297592)0.2368:0.12323940,(12human_NPPA:0.65314115,13platypus_NPPA:0.48629313)1.0000:0.44872189)0.0526:0.12028517,('14painted turtle NPPA-201 peptide: ENSCPBP00000031300 pep:protein coding':0.86408056,18human_NPPB:0.85570541)0.4737:0.12133540)0.0000:0.07282121,(15zebrafish_nppa:1.09677797,((23human_NPPC:0.68433746,24opossum_NPPC:0.54102654)0.9474:0.30572990,(25painted_turtle_NPPC:0.75556487,26spotted_gar_nppc:0.80257975)0.4737:0.07548124)0.6579:0.27235036)0.1053:0.01076593)0.0000:0.13292647,(5Trichinella_sp:1.42462292,17hagfish_CNP:1.41955946)0.0263:0.10396069)0.0000:0.01338934,(22spotted_gar_nppb:0.98882021,((7Strongylocentrotus_purpuratus:1.43388645,8Acanthaster_planci2:1.27416375)0.3421:0.07113046,(10Ciona_intestinalis:1.19978360,(6Acanthaster_planci:1.22233320,11Oikopleura_dioica2:1.20808526)0.2895:0.06491759)0.1579:0.10863262)0.0789:0.05580469)0.0263:0.05587402,(3Neodiprion_lecontei:1.26791697,(4Varroa_jacobsoni:1.32591911,(9Oikopleura_dioica:1.21922749,16lamprey_CNP:1.43669365)0.1579:0.09639877)0.0263:0.08105920)0.0000:0.07157206);

Results alignments

Lamprey cnp / Vollenhovia emeryi: id 19.1%, sim 36.1

Lamprey cnp /  Brazilian scorpion  38.5%, 53.8%

Lamprey cnp / Neodiprion lecontei   20.3%  30.2%

Lamprey cnp / Varroa jacobsoni 23.9%  33.0%

Lamprey cnp / Trichinella sp  21.1%  31.7%

Lamprey cnp / Acanthaster planci  22.0%  32.6%

Lamprey cnp / Strongylocentrotus purpuratus 27.6% 39.7%

Lamprey cnp / Oikopleura dioica 20.4% 35.2%

Lamprey cnp / Ciona intestinalis 26.6% 34.4%

Part 3. Docking analysis

Part 4. Binding site in vertebrates

NPR3 in fishes

Position of the binding amino acids in the alignment: 125, 153, 160, 178, 179, 186, 219, 223, 229, 233, 236, 249.

|  | % ID of the amino acids (with human) | % ID of the whole sequence (with human) |
| --- | --- | --- |
| Zebrafish | 83% | 52.0% |
| Spotted gar | 83% | 57.9% |
| Tetraodon | 75% | 55.5% |
| Fugu | 83% | 56.0% |

NPR1 in fishes

Position of the binding amino acids in the alignment: 114, 137, 140, 144, 160, 162, 169, 170, 204, 215, 216, 218, 219, 222, 223, 235

|  | % ID of the amino acids (with human) | % ID of the whole sequence (with human) |
| --- | --- | --- |
| Zebrafish | 31% | 61.8% |
| Spotted gar | 63% | 61.9% |
| Amazon molly | 44% | 59.3% |
| Platyfish | 44% | 57.5% |

NPR3 in Coelacanthe

Position of the binding amino acids in the alignment: 110, 138, 145, 163, 164, 171, 204, 208, 215, 219, 222, 234.  92% ID

NPR1 in Coelacanthe

Position of the binding amino acids in the alignment: 99, 122, 25, 129, 145, 147, 153, 154, 188, 199, 200, 202, 203, 206, 207, 220. 75% ID

Part 5. BLAST analysis

Vollenhovia emeryi

BLAST result

| [Max Score](https://blast.ncbi.nlm.nih.gov/Blast.cgi?CMD=Get&ALIGNMENTS=100&ALIGNMENT_VIEW=Pairwise&CDD_RID=DXCFTJP3014&CDD_SEARCH_STATE=0&DATABASE_SORT=0&DESCRIPTIONS=100&DYNAMIC_FORMAT=on&FIRST_QUERY_NUM=0&FORMAT_OBJECT=Alignment&FORMAT_PAGE_TARGET=&FORMAT_TYPE=HTML&GET_SEQUENCE=yes&I_THRESH=&LINE_LENGTH=60&MASK_CHAR=2&MASK_COLOR=1&NEW_VIEW=yes&NUM_OVERVIEW=100&PAGE=Proteins&QUERY_INDEX=0&QUERY_NUMBER=0&RESULTS_PAGE_TARGET=&RID=DXCFTNBK015&SHOW_LINKOUT=yes&SHOW_OVERVIEW=yes&STEP_NUMBER=&WORD_SIZE=6&OLD_VIEW=false&DISPLAY_SORT=1&HSP_SORT=1) | [Total Score](https://blast.ncbi.nlm.nih.gov/Blast.cgi?CMD=Get&ALIGNMENTS=100&ALIGNMENT_VIEW=Pairwise&CDD_RID=DXCFTJP3014&CDD_SEARCH_STATE=0&DATABASE_SORT=0&DESCRIPTIONS=100&DYNAMIC_FORMAT=on&FIRST_QUERY_NUM=0&FORMAT_OBJECT=Alignment&FORMAT_PAGE_TARGET=&FORMAT_TYPE=HTML&GET_SEQUENCE=yes&I_THRESH=&LINE_LENGTH=60&MASK_CHAR=2&MASK_COLOR=1&NEW_VIEW=yes&NUM_OVERVIEW=100&PAGE=Proteins&QUERY_INDEX=0&QUERY_NUMBER=0&RESULTS_PAGE_TARGET=&RID=DXCFTNBK015&SHOW_LINKOUT=yes&SHOW_OVERVIEW=yes&STEP_NUMBER=&WORD_SIZE=6&OLD_VIEW=false&DISPLAY_SORT=2&HSP_SORT=1) | [Query Cover](https://blast.ncbi.nlm.nih.gov/Blast.cgi?CMD=Get&ALIGNMENTS=100&ALIGNMENT_VIEW=Pairwise&CDD_RID=DXCFTJP3014&CDD_SEARCH_STATE=0&DATABASE_SORT=0&DESCRIPTIONS=100&DYNAMIC_FORMAT=on&FIRST_QUERY_NUM=0&FORMAT_OBJECT=Alignment&FORMAT_PAGE_TARGET=&FORMAT_TYPE=HTML&GET_SEQUENCE=yes&I_THRESH=&LINE_LENGTH=60&MASK_CHAR=2&MASK_COLOR=1&NEW_VIEW=yes&NUM_OVERVIEW=100&PAGE=Proteins&QUERY_INDEX=0&QUERY_NUMBER=0&RESULTS_PAGE_TARGET=&RID=DXCFTNBK015&SHOW_LINKOUT=yes&SHOW_OVERVIEW=yes&STEP_NUMBER=&WORD_SIZE=6&OLD_VIEW=false&DISPLAY_SORT=4&HSP_SORT=0) | [E value](https://blast.ncbi.nlm.nih.gov/Blast.cgi?CMD=Get&ALIGNMENTS=100&ALIGNMENT_VIEW=Pairwise&CDD_RID=DXCFTJP3014&CDD_SEARCH_STATE=0&DATABASE_SORT=0&DESCRIPTIONS=100&DYNAMIC_FORMAT=on&FIRST_QUERY_NUM=0&FORMAT_OBJECT=Alignment&FORMAT_PAGE_TARGET=&FORMAT_TYPE=HTML&GET_SEQUENCE=yes&I_THRESH=&LINE_LENGTH=60&MASK_CHAR=2&MASK_COLOR=1&NEW_VIEW=yes&NUM_OVERVIEW=100&PAGE=Proteins&QUERY_INDEX=0&QUERY_NUMBER=0&RESULTS_PAGE_TARGET=&RID=DXCFTNBK015&SHOW_LINKOUT=yes&SHOW_OVERVIEW=yes&STEP_NUMBER=&WORD_SIZE=6&OLD_VIEW=false&DISPLAY_SORT=0&HSP_SORT=0) | [Per. Ident](https://blast.ncbi.nlm.nih.gov/Blast.cgi?CMD=Get&ALIGNMENTS=100&ALIGNMENT_VIEW=Pairwise&CDD_RID=DXCFTJP3014&CDD_SEARCH_STATE=0&DATABASE_SORT=0&DESCRIPTIONS=100&DYNAMIC_FORMAT=on&FIRST_QUERY_NUM=0&FORMAT_OBJECT=Alignment&FORMAT_PAGE_TARGET=&FORMAT_TYPE=HTML&GET_SEQUENCE=yes&I_THRESH=&LINE_LENGTH=60&MASK_CHAR=2&MASK_COLOR=1&NEW_VIEW=yes&NUM_OVERVIEW=100&PAGE=Proteins&QUERY_INDEX=0&QUERY_NUMBER=0&RESULTS_PAGE_TARGET=&RID=DXCFTNBK015&SHOW_LINKOUT=yes&SHOW_OVERVIEW=yes&STEP_NUMBER=&WORD_SIZE=6&OLD_VIEW=false&DISPLAY_SORT=3&HSP_SORT=3) | Accession |
| --- | --- | --- | --- | --- | --- |

|  | 1415 | 1415 | 26% | 0.0 | 99.71% |
| --- | --- | --- | --- | --- | --- |

Protein function: Elongation factor, enzyme

Neodiprion lecontei

BLAST result

| [Max Score](https://blast.ncbi.nlm.nih.gov/Blast.cgi?CMD=Get&ALIGNMENTS=100&ALIGNMENT_VIEW=Pairwise&CDD_RID=DXD58HP1014&CDD_SEARCH_STATE=0&DATABASE_SORT=0&DESCRIPTIONS=100&DYNAMIC_FORMAT=on&FIRST_QUERY_NUM=0&FORMAT_OBJECT=Alignment&FORMAT_PAGE_TARGET=&FORMAT_TYPE=HTML&GET_SEQUENCE=yes&I_THRESH=&LINE_LENGTH=60&MASK_CHAR=2&MASK_COLOR=1&NEW_VIEW=yes&NUM_OVERVIEW=100&PAGE=Proteins&QUERY_INDEX=0&QUERY_NUMBER=0&RESULTS_PAGE_TARGET=&RID=DXD58EW1014&SHOW_LINKOUT=yes&SHOW_OVERVIEW=yes&STEP_NUMBER=&WORD_SIZE=6&OLD_VIEW=false&DISPLAY_SORT=1&HSP_SORT=1) | [Total Score](https://blast.ncbi.nlm.nih.gov/Blast.cgi?CMD=Get&ALIGNMENTS=100&ALIGNMENT_VIEW=Pairwise&CDD_RID=DXD58HP1014&CDD_SEARCH_STATE=0&DATABASE_SORT=0&DESCRIPTIONS=100&DYNAMIC_FORMAT=on&FIRST_QUERY_NUM=0&FORMAT_OBJECT=Alignment&FORMAT_PAGE_TARGET=&FORMAT_TYPE=HTML&GET_SEQUENCE=yes&I_THRESH=&LINE_LENGTH=60&MASK_CHAR=2&MASK_COLOR=1&NEW_VIEW=yes&NUM_OVERVIEW=100&PAGE=Proteins&QUERY_INDEX=0&QUERY_NUMBER=0&RESULTS_PAGE_TARGET=&RID=DXD58EW1014&SHOW_LINKOUT=yes&SHOW_OVERVIEW=yes&STEP_NUMBER=&WORD_SIZE=6&OLD_VIEW=false&DISPLAY_SORT=2&HSP_SORT=1) | [Query Cover](https://blast.ncbi.nlm.nih.gov/Blast.cgi?CMD=Get&ALIGNMENTS=100&ALIGNMENT_VIEW=Pairwise&CDD_RID=DXD58HP1014&CDD_SEARCH_STATE=0&DATABASE_SORT=0&DESCRIPTIONS=100&DYNAMIC_FORMAT=on&FIRST_QUERY_NUM=0&FORMAT_OBJECT=Alignment&FORMAT_PAGE_TARGET=&FORMAT_TYPE=HTML&GET_SEQUENCE=yes&I_THRESH=&LINE_LENGTH=60&MASK_CHAR=2&MASK_COLOR=1&NEW_VIEW=yes&NUM_OVERVIEW=100&PAGE=Proteins&QUERY_INDEX=0&QUERY_NUMBER=0&RESULTS_PAGE_TARGET=&RID=DXD58EW1014&SHOW_LINKOUT=yes&SHOW_OVERVIEW=yes&STEP_NUMBER=&WORD_SIZE=6&OLD_VIEW=false&DISPLAY_SORT=4&HSP_SORT=0) | [E value](https://blast.ncbi.nlm.nih.gov/Blast.cgi?CMD=Get&ALIGNMENTS=100&ALIGNMENT_VIEW=Pairwise&CDD_RID=DXD58HP1014&CDD_SEARCH_STATE=0&DATABASE_SORT=0&DESCRIPTIONS=100&DYNAMIC_FORMAT=on&FIRST_QUERY_NUM=0&FORMAT_OBJECT=Alignment&FORMAT_PAGE_TARGET=&FORMAT_TYPE=HTML&GET_SEQUENCE=yes&I_THRESH=&LINE_LENGTH=60&MASK_CHAR=2&MASK_COLOR=1&NEW_VIEW=yes&NUM_OVERVIEW=100&PAGE=Proteins&QUERY_INDEX=0&QUERY_NUMBER=0&RESULTS_PAGE_TARGET=&RID=DXD58EW1014&SHOW_LINKOUT=yes&SHOW_OVERVIEW=yes&STEP_NUMBER=&WORD_SIZE=6&OLD_VIEW=false&DISPLAY_SORT=0&HSP_SORT=0) | [Per. Ident](https://blast.ncbi.nlm.nih.gov/Blast.cgi?CMD=Get&ALIGNMENTS=100&ALIGNMENT_VIEW=Pairwise&CDD_RID=DXD58HP1014&CDD_SEARCH_STATE=0&DATABASE_SORT=0&DESCRIPTIONS=100&DYNAMIC_FORMAT=on&FIRST_QUERY_NUM=0&FORMAT_OBJECT=Alignment&FORMAT_PAGE_TARGET=&FORMAT_TYPE=HTML&GET_SEQUENCE=yes&I_THRESH=&LINE_LENGTH=60&MASK_CHAR=2&MASK_COLOR=1&NEW_VIEW=yes&NUM_OVERVIEW=100&PAGE=Proteins&QUERY_INDEX=0&QUERY_NUMBER=0&RESULTS_PAGE_TARGET=&RID=DXD58EW1014&SHOW_LINKOUT=yes&SHOW_OVERVIEW=yes&STEP_NUMBER=&WORD_SIZE=6&OLD_VIEW=false&DISPLAY_SORT=3&HSP_SORT=3) | Accession |
| --- | --- | --- | --- | --- | --- |

|  | 1526 | 1526 | 99% | 0.0 | 84.23% | [XP_012256658.1](https://www.ncbi.nlm.nih.gov/protein/XP_012256658.1?report=genbank&log$=prottop&blast_rank=2&RID=DXD58EW1014) |
| --- | --- | --- | --- | --- | --- | --- |

Protein function: RAS signaling antagonist

Varroa jacobsoni

BLAST result

| [Max Score](https://blast.ncbi.nlm.nih.gov/Blast.cgi?CMD=Get&ALIGNMENTS=100&ALIGNMENT_VIEW=Pairwise&CDD_RID=DXDNH5HU014&CDD_SEARCH_STATE=0&DATABASE_SORT=0&DESCRIPTIONS=100&DYNAMIC_FORMAT=on&FIRST_QUERY_NUM=0&FORMAT_OBJECT=Alignment&FORMAT_PAGE_TARGET=&FORMAT_TYPE=HTML&GET_SEQUENCE=yes&I_THRESH=&LINE_LENGTH=60&MASK_CHAR=2&MASK_COLOR=1&NEW_VIEW=yes&NUM_OVERVIEW=100&PAGE=Proteins&QUERY_INDEX=0&QUERY_NUMBER=0&RESULTS_PAGE_TARGET=&RID=DXDNHGFK015&SHOW_LINKOUT=yes&SHOW_OVERVIEW=yes&STEP_NUMBER=&WORD_SIZE=6&OLD_VIEW=false&DISPLAY_SORT=1&HSP_SORT=1) | [Total Score](https://blast.ncbi.nlm.nih.gov/Blast.cgi?CMD=Get&ALIGNMENTS=100&ALIGNMENT_VIEW=Pairwise&CDD_RID=DXDNH5HU014&CDD_SEARCH_STATE=0&DATABASE_SORT=0&DESCRIPTIONS=100&DYNAMIC_FORMAT=on&FIRST_QUERY_NUM=0&FORMAT_OBJECT=Alignment&FORMAT_PAGE_TARGET=&FORMAT_TYPE=HTML&GET_SEQUENCE=yes&I_THRESH=&LINE_LENGTH=60&MASK_CHAR=2&MASK_COLOR=1&NEW_VIEW=yes&NUM_OVERVIEW=100&PAGE=Proteins&QUERY_INDEX=0&QUERY_NUMBER=0&RESULTS_PAGE_TARGET=&RID=DXDNHGFK015&SHOW_LINKOUT=yes&SHOW_OVERVIEW=yes&STEP_NUMBER=&WORD_SIZE=6&OLD_VIEW=false&DISPLAY_SORT=2&HSP_SORT=1) | [Query Cover](https://blast.ncbi.nlm.nih.gov/Blast.cgi?CMD=Get&ALIGNMENTS=100&ALIGNMENT_VIEW=Pairwise&CDD_RID=DXDNH5HU014&CDD_SEARCH_STATE=0&DATABASE_SORT=0&DESCRIPTIONS=100&DYNAMIC_FORMAT=on&FIRST_QUERY_NUM=0&FORMAT_OBJECT=Alignment&FORMAT_PAGE_TARGET=&FORMAT_TYPE=HTML&GET_SEQUENCE=yes&I_THRESH=&LINE_LENGTH=60&MASK_CHAR=2&MASK_COLOR=1&NEW_VIEW=yes&NUM_OVERVIEW=100&PAGE=Proteins&QUERY_INDEX=0&QUERY_NUMBER=0&RESULTS_PAGE_TARGET=&RID=DXDNHGFK015&SHOW_LINKOUT=yes&SHOW_OVERVIEW=yes&STEP_NUMBER=&WORD_SIZE=6&OLD_VIEW=false&DISPLAY_SORT=4&HSP_SORT=0) | [E value](https://blast.ncbi.nlm.nih.gov/Blast.cgi?CMD=Get&ALIGNMENTS=100&ALIGNMENT_VIEW=Pairwise&CDD_RID=DXDNH5HU014&CDD_SEARCH_STATE=0&DATABASE_SORT=0&DESCRIPTIONS=100&DYNAMIC_FORMAT=on&FIRST_QUERY_NUM=0&FORMAT_OBJECT=Alignment&FORMAT_PAGE_TARGET=&FORMAT_TYPE=HTML&GET_SEQUENCE=yes&I_THRESH=&LINE_LENGTH=60&MASK_CHAR=2&MASK_COLOR=1&NEW_VIEW=yes&NUM_OVERVIEW=100&PAGE=Proteins&QUERY_INDEX=0&QUERY_NUMBER=0&RESULTS_PAGE_TARGET=&RID=DXDNHGFK015&SHOW_LINKOUT=yes&SHOW_OVERVIEW=yes&STEP_NUMBER=&WORD_SIZE=6&OLD_VIEW=false&DISPLAY_SORT=0&HSP_SORT=0) | [Per. Ident](https://blast.ncbi.nlm.nih.gov/Blast.cgi?CMD=Get&ALIGNMENTS=100&ALIGNMENT_VIEW=Pairwise&CDD_RID=DXDNH5HU014&CDD_SEARCH_STATE=0&DATABASE_SORT=0&DESCRIPTIONS=100&DYNAMIC_FORMAT=on&FIRST_QUERY_NUM=0&FORMAT_OBJECT=Alignment&FORMAT_PAGE_TARGET=&FORMAT_TYPE=HTML&GET_SEQUENCE=yes&I_THRESH=&LINE_LENGTH=60&MASK_CHAR=2&MASK_COLOR=1&NEW_VIEW=yes&NUM_OVERVIEW=100&PAGE=Proteins&QUERY_INDEX=0&QUERY_NUMBER=0&RESULTS_PAGE_TARGET=&RID=DXDNHGFK015&SHOW_LINKOUT=yes&SHOW_OVERVIEW=yes&STEP_NUMBER=&WORD_SIZE=6&OLD_VIEW=false&DISPLAY_SORT=3&HSP_SORT=3) | Accession |
| --- | --- | --- | --- | --- | --- |

| 58.5 | 58.5 | 89% | 1e-05 | 23.72% | [XP_008474842.1](https://www.ncbi.nlm.nih.gov/protein/XP_008474842.1?report=genbank&log$=prottop&blast_rank=5&RID=DXDNHGFK015) |
| --- | --- | --- | --- | --- | --- |

https://blast.ncbi.nlm.nih.gov/Blast.cgi#alnHdr_XP_008474842

Protein function: homolog maelstrom protein

Trichinella sp

BLAST result

| [Max Score](https://blast.ncbi.nlm.nih.gov/Blast.cgi?CMD=Get&ALIGNMENTS=100&ALIGNMENT_VIEW=Pairwise&CDD_RID=cdd_no_hits&CDD_SEARCH_STATE=1&DATABASE_SORT=0&DESCRIPTIONS=100&DYNAMIC_FORMAT=on&FIRST_QUERY_NUM=0&FORMAT_OBJECT=Alignment&FORMAT_PAGE_TARGET=&FORMAT_TYPE=HTML&GET_SEQUENCE=yes&I_THRESH=&LINE_LENGTH=60&MASK_CHAR=2&MASK_COLOR=1&NEW_VIEW=yes&NUM_OVERVIEW=100&PAGE=Proteins&QUERY_INDEX=0&QUERY_NUMBER=0&RESULTS_PAGE_TARGET=&RID=DXDYTRMS015&SHOW_LINKOUT=yes&SHOW_OVERVIEW=yes&STEP_NUMBER=&WORD_SIZE=6&OLD_VIEW=false&DISPLAY_SORT=1&HSP_SORT=1) | [Total Score](https://blast.ncbi.nlm.nih.gov/Blast.cgi?CMD=Get&ALIGNMENTS=100&ALIGNMENT_VIEW=Pairwise&CDD_RID=cdd_no_hits&CDD_SEARCH_STATE=1&DATABASE_SORT=0&DESCRIPTIONS=100&DYNAMIC_FORMAT=on&FIRST_QUERY_NUM=0&FORMAT_OBJECT=Alignment&FORMAT_PAGE_TARGET=&FORMAT_TYPE=HTML&GET_SEQUENCE=yes&I_THRESH=&LINE_LENGTH=60&MASK_CHAR=2&MASK_COLOR=1&NEW_VIEW=yes&NUM_OVERVIEW=100&PAGE=Proteins&QUERY_INDEX=0&QUERY_NUMBER=0&RESULTS_PAGE_TARGET=&RID=DXDYTRMS015&SHOW_LINKOUT=yes&SHOW_OVERVIEW=yes&STEP_NUMBER=&WORD_SIZE=6&OLD_VIEW=false&DISPLAY_SORT=2&HSP_SORT=1) | [Query Cover](https://blast.ncbi.nlm.nih.gov/Blast.cgi?CMD=Get&ALIGNMENTS=100&ALIGNMENT_VIEW=Pairwise&CDD_RID=cdd_no_hits&CDD_SEARCH_STATE=1&DATABASE_SORT=0&DESCRIPTIONS=100&DYNAMIC_FORMAT=on&FIRST_QUERY_NUM=0&FORMAT_OBJECT=Alignment&FORMAT_PAGE_TARGET=&FORMAT_TYPE=HTML&GET_SEQUENCE=yes&I_THRESH=&LINE_LENGTH=60&MASK_CHAR=2&MASK_COLOR=1&NEW_VIEW=yes&NUM_OVERVIEW=100&PAGE=Proteins&QUERY_INDEX=0&QUERY_NUMBER=0&RESULTS_PAGE_TARGET=&RID=DXDYTRMS015&SHOW_LINKOUT=yes&SHOW_OVERVIEW=yes&STEP_NUMBER=&WORD_SIZE=6&OLD_VIEW=false&DISPLAY_SORT=4&HSP_SORT=0) | [E value](https://blast.ncbi.nlm.nih.gov/Blast.cgi?CMD=Get&ALIGNMENTS=100&ALIGNMENT_VIEW=Pairwise&CDD_RID=cdd_no_hits&CDD_SEARCH_STATE=1&DATABASE_SORT=0&DESCRIPTIONS=100&DYNAMIC_FORMAT=on&FIRST_QUERY_NUM=0&FORMAT_OBJECT=Alignment&FORMAT_PAGE_TARGET=&FORMAT_TYPE=HTML&GET_SEQUENCE=yes&I_THRESH=&LINE_LENGTH=60&MASK_CHAR=2&MASK_COLOR=1&NEW_VIEW=yes&NUM_OVERVIEW=100&PAGE=Proteins&QUERY_INDEX=0&QUERY_NUMBER=0&RESULTS_PAGE_TARGET=&RID=DXDYTRMS015&SHOW_LINKOUT=yes&SHOW_OVERVIEW=yes&STEP_NUMBER=&WORD_SIZE=6&OLD_VIEW=false&DISPLAY_SORT=0&HSP_SORT=0) | [Per. Ident](https://blast.ncbi.nlm.nih.gov/Blast.cgi?CMD=Get&ALIGNMENTS=100&ALIGNMENT_VIEW=Pairwise&CDD_RID=cdd_no_hits&CDD_SEARCH_STATE=1&DATABASE_SORT=0&DESCRIPTIONS=100&DYNAMIC_FORMAT=on&FIRST_QUERY_NUM=0&FORMAT_OBJECT=Alignment&FORMAT_PAGE_TARGET=&FORMAT_TYPE=HTML&GET_SEQUENCE=yes&I_THRESH=&LINE_LENGTH=60&MASK_CHAR=2&MASK_COLOR=1&NEW_VIEW=yes&NUM_OVERVIEW=100&PAGE=Proteins&QUERY_INDEX=0&QUERY_NUMBER=0&RESULTS_PAGE_TARGET=&RID=DXDYTRMS015&SHOW_LINKOUT=yes&SHOW_OVERVIEW=yes&STEP_NUMBER=&WORD_SIZE=6&OLD_VIEW=false&DISPLAY_SORT=3&HSP_SORT=3) | Accession |
| --- | --- | --- | --- | --- | --- |

|  | 106 | 106 | 18% | 5e-22 | 94.23% | [KRY56513.1](https://www.ncbi.nlm.nih.gov/protein/KRY56513.1?report=genbank&log$=prottop&blast_rank=8&RID=DXDYTRMS015) |
| --- | --- | --- | --- | --- | --- | --- |

Protein function: Enzyme, catalyse the conversion of RNA retro elements

Acanthaster planci

BLAST result

| [Max Score](https://blast.ncbi.nlm.nih.gov/Blast.cgi?CMD=Get&ALIGNMENTS=100&ALIGNMENT_VIEW=Pairwise&CDD_RID=DXE7T1XV015&CDD_SEARCH_STATE=0&DATABASE_SORT=0&DESCRIPTIONS=100&DYNAMIC_FORMAT=on&FIRST_QUERY_NUM=0&FORMAT_OBJECT=Alignment&FORMAT_PAGE_TARGET=&FORMAT_TYPE=HTML&GET_SEQUENCE=yes&I_THRESH=&LINE_LENGTH=60&MASK_CHAR=2&MASK_COLOR=1&NEW_VIEW=yes&NUM_OVERVIEW=100&PAGE=Proteins&QUERY_INDEX=0&QUERY_NUMBER=0&RESULTS_PAGE_TARGET=&RID=DXE7TFA0014&SHOW_LINKOUT=yes&SHOW_OVERVIEW=yes&STEP_NUMBER=&WORD_SIZE=6&OLD_VIEW=false&DISPLAY_SORT=1&HSP_SORT=1) | [Total Score](https://blast.ncbi.nlm.nih.gov/Blast.cgi?CMD=Get&ALIGNMENTS=100&ALIGNMENT_VIEW=Pairwise&CDD_RID=DXE7T1XV015&CDD_SEARCH_STATE=0&DATABASE_SORT=0&DESCRIPTIONS=100&DYNAMIC_FORMAT=on&FIRST_QUERY_NUM=0&FORMAT_OBJECT=Alignment&FORMAT_PAGE_TARGET=&FORMAT_TYPE=HTML&GET_SEQUENCE=yes&I_THRESH=&LINE_LENGTH=60&MASK_CHAR=2&MASK_COLOR=1&NEW_VIEW=yes&NUM_OVERVIEW=100&PAGE=Proteins&QUERY_INDEX=0&QUERY_NUMBER=0&RESULTS_PAGE_TARGET=&RID=DXE7TFA0014&SHOW_LINKOUT=yes&SHOW_OVERVIEW=yes&STEP_NUMBER=&WORD_SIZE=6&OLD_VIEW=false&DISPLAY_SORT=2&HSP_SORT=1) | [Query Cover](https://blast.ncbi.nlm.nih.gov/Blast.cgi?CMD=Get&ALIGNMENTS=100&ALIGNMENT_VIEW=Pairwise&CDD_RID=DXE7T1XV015&CDD_SEARCH_STATE=0&DATABASE_SORT=0&DESCRIPTIONS=100&DYNAMIC_FORMAT=on&FIRST_QUERY_NUM=0&FORMAT_OBJECT=Alignment&FORMAT_PAGE_TARGET=&FORMAT_TYPE=HTML&GET_SEQUENCE=yes&I_THRESH=&LINE_LENGTH=60&MASK_CHAR=2&MASK_COLOR=1&NEW_VIEW=yes&NUM_OVERVIEW=100&PAGE=Proteins&QUERY_INDEX=0&QUERY_NUMBER=0&RESULTS_PAGE_TARGET=&RID=DXE7TFA0014&SHOW_LINKOUT=yes&SHOW_OVERVIEW=yes&STEP_NUMBER=&WORD_SIZE=6&OLD_VIEW=false&DISPLAY_SORT=4&HSP_SORT=0) | [E value](https://blast.ncbi.nlm.nih.gov/Blast.cgi?CMD=Get&ALIGNMENTS=100&ALIGNMENT_VIEW=Pairwise&CDD_RID=DXE7T1XV015&CDD_SEARCH_STATE=0&DATABASE_SORT=0&DESCRIPTIONS=100&DYNAMIC_FORMAT=on&FIRST_QUERY_NUM=0&FORMAT_OBJECT=Alignment&FORMAT_PAGE_TARGET=&FORMAT_TYPE=HTML&GET_SEQUENCE=yes&I_THRESH=&LINE_LENGTH=60&MASK_CHAR=2&MASK_COLOR=1&NEW_VIEW=yes&NUM_OVERVIEW=100&PAGE=Proteins&QUERY_INDEX=0&QUERY_NUMBER=0&RESULTS_PAGE_TARGET=&RID=DXE7TFA0014&SHOW_LINKOUT=yes&SHOW_OVERVIEW=yes&STEP_NUMBER=&WORD_SIZE=6&OLD_VIEW=false&DISPLAY_SORT=0&HSP_SORT=0) | [Per. Ident](https://blast.ncbi.nlm.nih.gov/Blast.cgi?CMD=Get&ALIGNMENTS=100&ALIGNMENT_VIEW=Pairwise&CDD_RID=DXE7T1XV015&CDD_SEARCH_STATE=0&DATABASE_SORT=0&DESCRIPTIONS=100&DYNAMIC_FORMAT=on&FIRST_QUERY_NUM=0&FORMAT_OBJECT=Alignment&FORMAT_PAGE_TARGET=&FORMAT_TYPE=HTML&GET_SEQUENCE=yes&I_THRESH=&LINE_LENGTH=60&MASK_CHAR=2&MASK_COLOR=1&NEW_VIEW=yes&NUM_OVERVIEW=100&PAGE=Proteins&QUERY_INDEX=0&QUERY_NUMBER=0&RESULTS_PAGE_TARGET=&RID=DXE7TFA0014&SHOW_LINKOUT=yes&SHOW_OVERVIEW=yes&STEP_NUMBER=&WORD_SIZE=6&OLD_VIEW=false&DISPLAY_SORT=3&HSP_SORT=3) | Accession |
| --- | --- | --- | --- | --- | --- |

|  | 155 | 155 | 25% | 2e-39 | 47.89% | [PIK40037.1](https://www.ncbi.nlm.nih.gov/protein/PIK40037.1?report=genbank&log$=prottop&blast_rank=7&RID=DXE7TFA0014) |
| --- | --- | --- | --- | --- | --- | --- |

		

Protein function: [putative zinc finger CCHC domain-containing protein 3-like](https://blast.ncbi.nlm.nih.gov/Blast.cgi#alnHdr_PIK40037)

Strongylocentrotus purpuratus

Uncharacterised

Oikopleura dioica

Uncharacterised

Ciona intestinalis

Protein function: Enzyme, regulation of cell survival
